# Identification of myeloid derived suppressor cells in the peripheral blood of tumor bearing dogs

**DOI:** 10.1186/1746-6148-8-209

**Published:** 2012-10-31

**Authors:** Matthew Sherger, William Kisseberth, Cheryl London, Susan Olivo-Marston, Tracey L Papenfuss

**Affiliations:** 1 Department of Veterinary Biosciences, College of Veterinary Medicine, The Ohio State University, 1900 Coffey Road, Columbus, OH, USA; 2 Department of Veterinary Clinical Sciences, College of Veterinary Medicine, The Ohio State University, 1900 Coffey Road, Columbus, OH, USA; 3 Division of Epidemiology, College of Public Health, The Ohio State University, 1841 Neil Avenue, Columbus, OH, USA

**Keywords:** Myeloid derived suppressor cells, Cancer immunotherapy, Immunosuppression

## Abstract

**Background:**

Myeloid derived suppressor cells (MDSCs) are a recently described population of immune cells that significantly contribute to the immunosuppression seen in cancer patients. MDSCs are one of the most important factors that limit the efficacy of cancer immunotherapy (e.g. cancer vaccines) and MDSC levels are increased in cancer in multiple species. Identifying and targeting MDSCs is actively being investigated in the field of human oncology and is increasingly being investigated in veterinary oncology. The treatment of canine cancer not only benefits dogs, but is being used for translational studies evaluating and modifcying candidate therapies for use in humans. Thus, it is necessary to understand the immune alterations seen in canine cancer patients which, to date, have been relatively limited. This study investigates the use of commercially available canine antibodies to detect an immunosuppressive (CD11b^low^/CADO48^low^) cell population that is increased in the peripheral blood of tumor-bearing dogs.

**Results:**

Commercially available canine antibodies CD11b and CADO48A were used to evaluate white blood cells from the peripheral blood cells of forty healthy control dogs and forty untreated, tumor-bearing dogs. Tumor-bearing dogs had a statistically significant increase in CD11b^low^/CADO48A^low^ cells (7.9%) as compared to the control dogs (3.6%). Additionally, sorted CD11b^low^/CADO48A^low^ generated *in vitro* suppressed the proliferation of canine lymphocytes.

**Conclusions:**

The purpose of this study was aimed at identifying potential canine specific markers for identifying MDSCs in the peripheral blood circulation of dogs. This study demonstrates an increase in a unique CD11b^low^/CADO48A^low^ cell population in tumor-bearing dogs. This immunophenotype is consistent with described phenotypes of MDSCs in other species (i.e. mice) and utilizes commercially available canine-specific antibodies. Importantly, CD11b^low^/CADO48A^low^ from a tumor environment suppress the proliferation of lymphocytes. These results provide a useful phenotype of cells increased in canine cancer patients that may serve as a useful prognostic marker for assessing immune status and functional response to cancer immunotherapies in dogs. Understanding MDSCs in dogs will allow for increased effectiveness of cancer immunotherapy in both dogs and humans.

## Background

Myeloid derived suppressor cells (MDSC) are immature myeloid cells produced by bone marrow precursor cells that are increased in a variety of diseases. Most significantly, MDSCs are increased in cancer patients and significantly contribute to the immunosuppression of these cancer patients [1]. MDSCs are immature myeloid cells which are arrested during differentiation (myelopoiesis) and accumulate [2,3]. Utilizing the immune system to specifically target the destruction of cancer cells (i.e. cancer immunotherapy) is conceptually appealing but has proven to be difficult therapeutically. The efficacy of cancer vaccines has proven relatively unsuccessful in patient populations due, in large part, to the dysregulated immune system of cancer patients. MDSCs are significant contributors to the immunosuppression in cancer. Although suppressive myeloid cells were recognized over forty years ago, the understanding that MDSCs contribute to the immunosuppression that limits cancer immunotherapeutics has renewed the interest in these immunosuppressive myeloid cells [4-6].

MDSCs are a heterogeneous population of cells with a variety of phenotypic markers being recognized which have variably been used to identify subsets. By and large, MDSCs are identified as CD11b^+^Gr1^+^ in mice and CD33^+^HLA^–^DR^–^Lin^–^ in humans although numerous additional markers (e.g. S100A, etc.) have been used to categorize MDSC subsets. The dual expression of CD11b (myeloid marker) and Gr1 (granulocytic marker) highlights the immature nature of these cells and the fact that these cells arise from a common myeloid precursor that differentiates into dendritic cells (DCs), macrophages and granulocytes. Although present normally at low levels, under pathologic conditions, elevated numbers of these cells are found in both the peripheral circulation and lymphoid organs [7]. The accumulation of MDSCs is thought to be due to a variety of factors including a wide array of soluble factors produced from the tumor environment (e.g. GM-CSF, VEGF, IL-1beta, IL-6, S100A8/A9, etc.). A variety of therapies aimed at limiting MDSC actions have been used to prevent the formation of MDSCs (e.g. tyrosine kinase inhibitors, Sunitinib), reduce MDSC accumulation (e.g. gemcitabine, 5-fluorouracil), affect MDSCs inhibitory abilities (e.g. phosphodiesterase 5 inhibitors, nitroaspirin) or promote MDSC differentiation (e.g. retinoic acid and vitamin D) [1,8-14].

MDSCs suppress both innate and adaptive immune responses through a combination of cell contact-mediated mechanisms (e.g. expression of inhibitory surface marker PD-L1) and the production of a wide array of soluble mediators (e.g. arginase, nitric oxide and reactive oxygen species) [15,16]. Cells which have been shown to be inhibited by MDSCs include natural killer (NK) cells, macrophages, DCs, CD4^+^ helper T cells, CD8^+^ (cytotoxic) T cells and NK-T cells. Additionally, MDSCs are recognized to promote regulatory T (Treg) cell production where Tregs are potently immunosuppressive in their own right [17]. Through these multiple actions, MDSCs significantly contribute to the immune dysregulation and immunosuppression seen during cancer and are important roadblocks to achieving complete anti-tumor immunity.

Assessment of MDSC levels in cancer patients may provide an important means to evaluate not only relative immune status, but also may be useful biomarkers to evaluate response to therapy. For that reason, easy, rapid and accurate identification of MDSCs is critical. CD11b^+^Gr1^+^ cells are useful markers used to identify MDSCs in mice and studies have demonstrated specific monocytic (Ly6C^hi^Ly6G^+^) and granulocytic (Ly6C^low^Ly6G^+^) subsets that may be distinctly contribute to the immunosuppression present in cancer [1,18,19]. Similarly, in humans, the CD33^+^HLA^–^DR^–^Lin^–^ MDSC population can be categorized into subpopulations but the contribution of specific subsets to disease pathogenesis is less well defined than in mouse models. The relative identification of MDSCs in other species remains relatively undescribed but has important therapeutic applications in both veterinary and human oncology.

Increasingly, the dog is being used as both a large animal model for carcinogenesis studies and the assessment of cancer therapies due to the similarities between dogs and humans (e.g. outbred population, shared environmental exposures, etc.). Additionally, dog owners are increasingly interested in state-of-the-art therapeutics and are receptive to enrolling their dogs into clinical trials for experimental therapeutics. Thus, there are increasing opportunities not only to assess the responsiveness of canine cancer patients to particular novel therapeutics and cancer immunotherapy, but to apply the acquired information (e.g. drug pharmacokinetics, pharmacodynamics, dosing regimens and efficacy) for clinical applications in human cancer patients. At present, the use of cancer immunotherapy is relatively limited in veterinary medicine but the approval and clinical application of immunotherapies such as the canine melanoma vaccine and Palladia for canine mast cells are reflective of an increased interest in this useful therapeutic approach [9,20]. Given these facts, it becomes necessary to understand the immune alterations seen in cancer patients which, to date, have been relatively limited [21,22]. The accurate identification and assessment of MDSC levels in dogs is important to understand the contribution of these potent immunosuppressive cells in canine cancer. Additionally, the identification of an MDSC phenotype provides a means to assess peripheral blood MDSCs which may serve as a useful prognostic marker for assessing immune status and functional response to cancer immunotherapies. This study identified canine specific markers that can be used to identify specific myeloid cell populations within clinical samples from dogs. Our data demonstrate that canine-specific antibodies can be used to identify a specific population of myeloid cells (CD11b^low^CADO48A^low^) which are increased in tumor-bearing canine patients and that purified CD11b^low^CADO48A^low^ cells suppressed the proliferation of canine lymphocytes.

## Methods

### Study Design/Animals

Eighty client-owned dogs were prospectively enrolled in the study at The Ohio State University’s Veterinary Medical Center. Forty dogs presenting to the Community Practice or Blood Bank Services without evidence or history of neoplasia were enrolled into the control group. Forty dogs presenting to the Medical or Radiation Oncology Services were enrolled into the experimental population. Inclusion criteria for patients in the experimental population were (1) cytological or histopatholgic diagnosis of a mesenchymal or epithelial neoplasm, (2) local or metastatic disease, (3) no prior surgical of chemotherapeutic treatment and (4) no prior history of neoplasia. No patients with round cell tumors were enrolled in the study. Animal use was approved by The Ohio State University’s Institutional Animal Care and Use Committee and The Ohio State University’s Veterinary Medical Center’s Clinical Research Advisory Committee.

### Flow Cytometry Staining and Optimization

**Figure 1:**
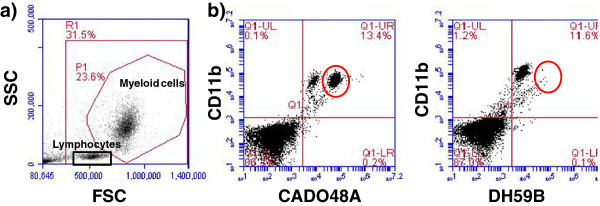
**Staining characteristics and evaluation of two commercially available canine granulocytic antibodies of canine peripheral blood. (a)** Cells were analyzed according to forward and side scatter. Cells were either gated to include all live and dead (no gate), all live cells including lymphocytes (R1, lymphocytes are circled) and all myeloid cells (P1). (**b**) Cells were labeled using canine anti-CD11b and either (**a**) CADO48A or (**b**) DH59B with the same concentration of secondary antibody FITC. As can be seen in (**a**), CADO48A allows visualization of an additional cell population as compared to DH59B (**b**). These results were repeatable with multiple blood samples from different dogs. The cells in (**b**) were gated on P1 as indicated in (**a**).

**Figure 2:**
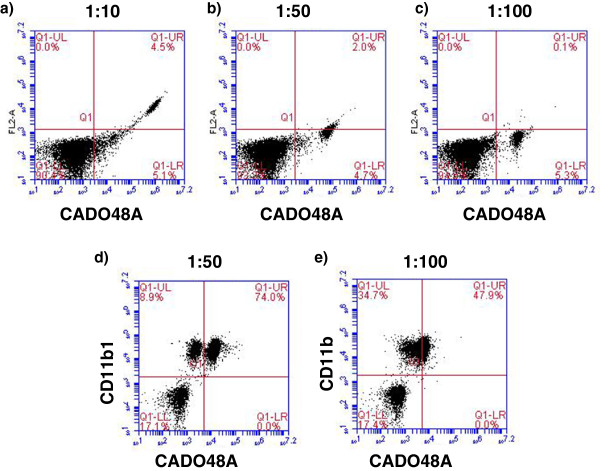
**Optimization of secondary antibody staining concentration for detection of CADO48A.** Cells were stained with the primary antibody CADO48A alone followed by (**a**) 1:10 (**b**) 1:50 and (**c**) 1:100 dilutions of a secondary FITC antibody. Optimal detection was seen between 1:50 and 1:100 dilution with CADO48A alone. Secondary FITC concentrations of 1:50 (**d**) and 1:100 (**e**) to detect CADO48A on pre-labeled CD11b cells were then evaluated and demonstrated that a 1:50 concentration of FITC was optimal for optimal detection of distinct CADO48A+ cells. Based on these findings, a 1:50 FITC concentration was used in all clinical samples. All cells in these diagrams were gated on P1.

Initial studies optimized the application of commercially available antibodies for detection of MDSCs in dogs. Whole blood samples were obtained through peripheral venipuncture from dogs using approved Institutional Animal Care and Use Committee (IACUC) protocols. The concentration of antibodies and the relative influence of sample handling and influence of time and fixation after staining were evaluated (Figure 1). Briefly, erythrocytes were removed from whole blood samples using erythrocyte lysis buffer (NH_4_Cl/KHCO_3_/EDTA), washed with 1x PBC, resuspended in fluorescence-activated cell sorting buffer (FACS; PBS containing 0.1% BSA and 0.1% sodium azide) and placed into flow tubes at a final concentration of 5x10^5^/100uL. Cells were then stained with a panel of antibodies including MHC class II (AbdSerotec, MCA1044F; clone YKIX334.2), IgG1 (AbdSerotec, MCA928), CD11b (AbdSerotec, MCA1777S; clone CA16.3E10), CD14 (AbdSerotec, MCA1568A647; clone TUK4), DH59B (previously VMRD, now Monoclonal Antibody Center, Washington State University; clone DH59B), CADO48A (Monoclonal Antibody Center, Washington State University; clone CADO48A) and available isotype controls. All antibodies were primary, non-conjugated with the exception of MHC class II and CD14 which were directly conjugated to fluorescein isothiocyanate (FITC) and Alexa Fluor 647 (BD Biosciences), respectively. Secondary antibodies of either phycoerythrin (PE; 5 uL) or Fluorescein isothiocyanate (FITC; 1uL) both from AbD Serotec were used to stain CD11b, CADO48A and DH59B at various dilutions (1:10, 1:50, and 1:100) for optimization studies. Results from these optimization studies were then used on the clinical patient samples. Samples were run on an BD Accuri flow cytometer and analyzed with BD Accuri CFlow analysis software. Samples were run immediately following staining or fixed in 4% formalin for 4, 24 and 48 hours after staining for optimization (Figure 2) and immediately upon preparation for clinical patient blood samples.

### Flow Cytometry of patient samples

**Figure 3:**
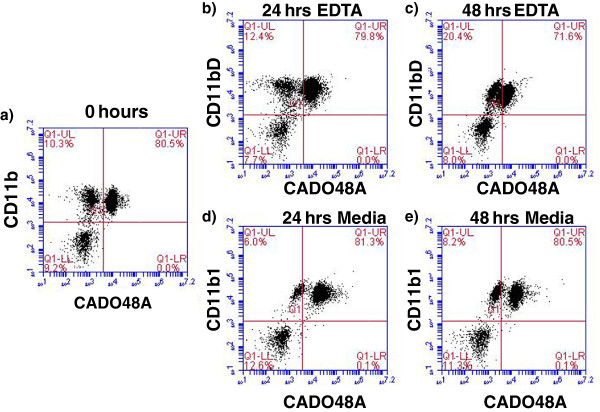
**Effects of EDTA versus media storage of patient blood samples on flow cytometric results.** Peripheral blood cells were (**a**) stained immediate following collection with CD11b and CADO48A or maintained in EDTA collection tubes for (**b**) 24 or (**c**) 48 hours or in media for (**d**) 24 or (**e**) 48 hours prior to staining with CD11b and CADO48A. Both EDTA and media samples were kept refrigerated. These findings show a decrease over time of population distinction in EDTA with minimal changes when cells are kept in media up to 48 hours. All cells were gated on P1.

**Figure 4:**
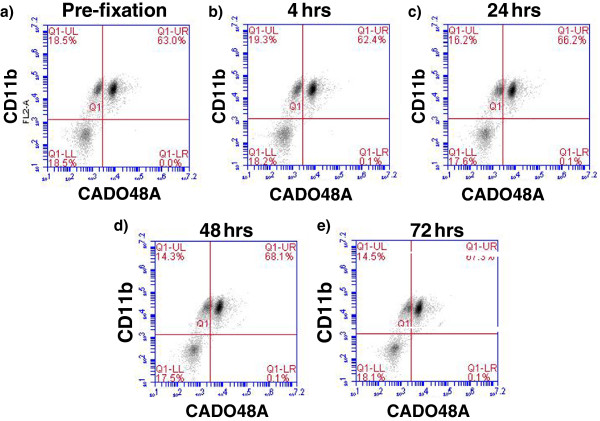
**Effections of fixation on CD11b and CADO48A expression levels.** Cells were stained for CD11b and CADO48A and evaluated pre-fixation (**a**) or subsequently fixed with 4% paraformaldehyde at (**b**) 4 hours, (**c**) 24 hours), (**d**) 48 hours or (**e**) 72 hours post-fixation before analysis on the flow cytometer. Fixation of cells appears to have limited effects on expression levels of CD11b and CADO48A expression up to 24 hours and then only mild increases of 4-6% of CD11b^+^CADO48A^+^ cells are seen at 24, 48 and 72 hours post-fixation. All cells seen in this diagram were gated on P1.

**Figure 5:**
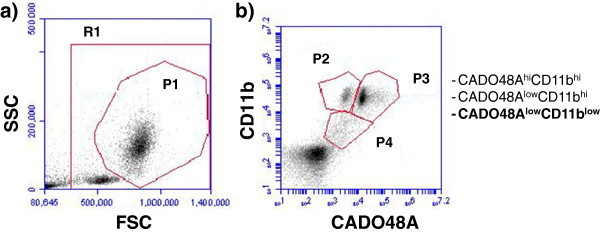
**Gated myeloid subpopulations evaluated for patient population.** All peripheral blood myeloid cells were evaluated using the P1 gate (non-lymphocytes) and subsequently gated based on relatively expression levels of CD11b and CADO48A where CADO48A^hi^/CD11b^hi^ (gate P2), CADO48A^low^/CD11b^hi^ (gate P3) and CADO48A^low^/CD11b^low^ (gate P4) likely represent granulocytes/neutrophils, monocytes and MDSCs, respectively.

Upon blood collection (EDTA tube), the sample was kept at 4°C before staining and all samples were processed within 12 hours of collection. Each patient sample was divided into sample evaluation of 1) cells alone (no antibody), 2) MHC class II, 3) IgG1, 4) CD11b, 5) CADO48A and 6) a combination of CD11b and CADO48A (Figure 3) using antibody concentrations determined during the optimization protocols. The primary antibodies CD11b and CADO were secondarily stained with 5uL PE and 1 uL of a 1:50 dilution of FITC respectively. As previously described, samples were prepared and analyzed with the BD Accuri flow cytometer. Data analysis of specific blood cell populations was performed. Specifically, cells were evaluated based on 1) all cells (both dead and live cells, ungated), 2) all peripheral blood mononuclear cells (PBMC) or R1, and 3) all non-lymphocytes based on the appearance of cells on forward scatter (FSC) and side scatter (SSC) or P1. Direct CD11b^+^CADO48A^+^ staining on these cell populations was assessed (Figure 4). Subsequent gates were assigned as follows P2 (CD11b^hi^CADO^hi^), P3 (CD11b^hi^CADO^low^) and P4 (CD11b^low^CADO^low^) (Figure 5). For all gates, the percent and total count of all cells staining positive for both antibodies was determined.

### *In vitro* differentiation, proliferation assay and cytospin of canine MDSCs

Canine bone marrow was approved from humanely euthanized dogs on an approved IACUC protocol. Bone marrow was differentiated in the presence of 10 ng/ml human GM-CSF (or 20 ng/ml canine GM-CSF) for 4–5 days with or without 20% tumor-conditioned media from a canine-specific melanoma MEL-16 line (kindly provided by Dr. Cheryl London). Cells were then labeled as described above for CD11b and CADO48A and sorted using a FACSAris flow sorter. Purified cells were then co-cultured at a 1:5 ratio with responder canine splenocytes and stimulated for 40 hours with 1 ug/ml LPS (Sigma) or 3 ug/ml conA (Sigma) with a final pulse of ^3^ H thymidine in the last 18 hours of culture. An aliquot of cells was prepared by cytospin (1500 rpm for 5 minutes), stained with Wright-Giemsa and photomicrographs taken at a 60x magnification.

### Statistical Analysis

For all statistical analyses, the percentage of cells staining positive for both CADO48A and CD11b were evaluated. Differences between the control and experimental groups were compared using a Wilcoxon rank-sum (Mann–Whitney) test. Additional comparisons between the individual tumor types (sarcoma, carcinoma and melanoma) were made using a Kruskal-Wallis equality-of-populations rank test. For all comparisons made, p-values less than 0.05 were considered to be significant.

## Results

### Flow Cytometry Optimization

Canine blood samples evaluated by forward scatter (FSC) and side scatter (SSC) (Figure 1) demonstrated distinct populations of small non-granular cells (i.e. lymphocytes) and large granular cells (P1). Based on size and granularity, large granular cells present within the P1 gate were evaluated for expression of cell surface markers of CD11b and commercially available canine granulocyte markers CADO48A and DH59B. Figure 1b demonstrates an increased distinction in cell subpopulations evidence with CADO48A staining that was not apparent with DH59B staining. Based on these results, we chose to utilize CADO48A in identifying potential canine MDSCs and myeloid cell populations in canine peripheral blood samples. We first optimized the secondary antibody concentration to detect double positive CD11b and CADO48A. Previous work (data not shown) showed that a 1:50 dilution for secondary antibody staining of CD11b effective identifies CD11b^+^ cells in canine peripheral blood. We next evaluated specific dilutions of secondary FITC antibody staining for detection of CADO48A. Figure 2 shows that a optimal detection was seen at a concentration between 1:50 and 1:100 secondary FITC antibody both in single-labeled CADO48A^+^ cells and in cells that were dual-labeled with CD11b on PE and CADO48A on FITC. Based on these findings, a 1:50 FITC concentration was used in all clinical samples. All cells in these diagrams were gated on P1.

Given the variable nature of procurement, handling and processing of clinical samples, we next evaluated the influence of sample handling, timing of antibody labeling and fixation of samples. Figure 3 shows the results of evaluating the effect of immediate staining of cells after collection or staining after storage in either EDTA or 5% RPMI culture media for 24 or 48 hours. Cells that were stained immediately upon collection demonstrated the most consistent results compared to those stored in EDTA or media for 24 hours. While cells stored in media demonstrated no change in CD11b/CADO48A expression after 48 hours, there was a slight decrease in CD11b^+^CADO48A^+^ cells in cells stored in EDTA for 48 hours. In general, the entire population of cells in 48 hour EDTA-treated cells had diminished CADO48A expression which contributed to the decreased in double positive cells. These findings suggest that media storage of samples for up to 48 hours can provide consistent and similar results to samples processed immediately for evaluated CD11b/CADO48A expression while storage of samples in EDTA for more than 24 hours may alter the receptor expression of cells. For all subsequent blood sample evaluation, samples were processed immediately unless otherwise indicated.

Fixation of cells with 4% paraformaldehyde following staining with flow antibodies demonstrated that the expression levels of CD11b and CADO48A remain relatively constant over time with no change evident in the percentage of CD11b^+^CADO48A^+^ cells seen even after 72 hours (Figure 4). While immediate evaluation of clinical samples for the expression levels of cell surface markers is ideal, our results suggest that samples can be stored for up to 24 hours in EDTA or 48 hours in media and that cells can be fixed following antibody staining and analyzed up to 72 hours later without dramatically impacting the expression of CD11b^+^CADO48^+^ levels.

### Clinical Patient Blood MDSC Evaluation

**Figure 6:**
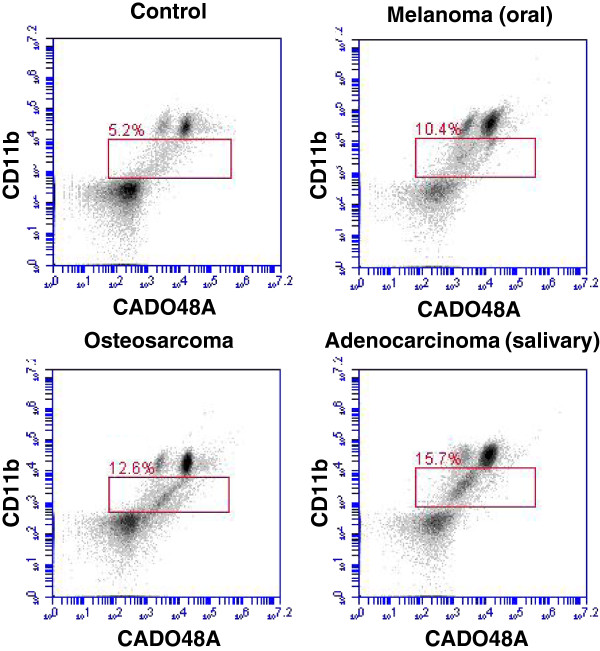
**Increased percentage of CD11b^low^/CADO48A^low^ in tumor-bearing canine patients.** The expression levels of CD11b^low^ and CADO48A^low^ was evaluated from the peripheral blood of representative canine patients and show an increase of CD11b^low^/CADO48^low^ in tumor-bearing canine patients.

**Figure 7:**
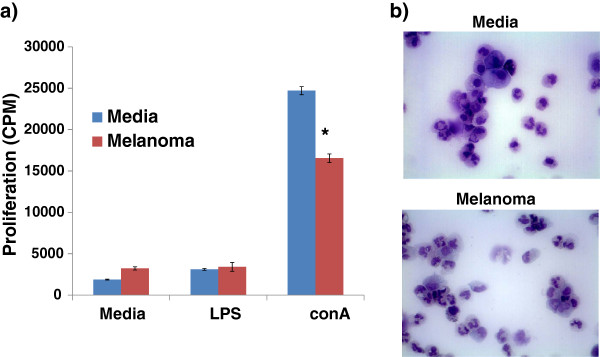
**Purified CD11b^low^CADO48A^low^ cells from in vitro differentiated myeloid cells suppressed the proliferation of T and B lymphocytes.** Bone marrow cells were differentiated in either GM-CSF (Media) or GM-CSF with canine melanoma tumor-conditioned media for 5 days. Cells were purified and co-cultured at a 1:5 ratio with responder canine splenocytes and the (**a**) proliferation of cells determined. A cytospin (**b**) of purified cells shows a mixed population of myeloid and granulocytic precursor cells demonstrating monocytic, granulocytic and ring-shaped nuclei are present in the CD11b^low/^CADO48A^low^ populations of both media and melanoma cells. Photomicrograph of the cytospin is at 60x magnification.

Following the optimization studies, we next evaluated the patient populations of both tumor-bearing and control dogs for the presence of MDSCs. A total of 80 patients were enrolled into the study between April 2011 and January 2012. The control group was comprised of 40 dogs with a median age of 5.0 years (range 3–15 years) and a variety of breeds represented as outlined in Table 1. The experimental tumor-bearing group was comprised of 40 dogs with a median age of 9.3 years (range 3–14 years) with represented breeds and various tumor types (sarcomas; n=19), carcinomas; n=18) and oral melanomas; n=3) represented (Table 1). The relative expression levels of CD11b and granulocytic markers, such as CADO48A, have been used to identify specific populations of MDSCs. Based on differing levels of both CD11b and CADO48A expression in CD11b^+^CADO48A^+^ cells we found 3 distinct cell populations (P2-P4) that are shown in Figure 6. These cells were CADO48A^hi^/CD11b^hi^ (P2), CADO48A^low^/CD11b^hi^ (P3) and CADO48A^low^/CD11b^low^ (P4) and, based on staining characteristics of CD11b and granulocytic marker expression, are most consistent with a neutrophil, monocyte and myeloid precursor (i.e. putative MDSC) population, respectively. To determine whether specific myeloid cell populations are increased in tumor-bearing dogs, we next evaluated the levels of P2, P3 and P4 in tumor-bearing versus control dogs. Table 2 demonstrates the percentage of positive cells and associated statistical parameters (e.g. mean, median, SD, min, max) for both CD11b and CADO48A in control and tumor-bearing dogs. While no significant differences were seen in either P2 or P3, a statistically significant increase in CD11b^low^/CADO48A^low^ population (i.e. P4 gate) was seen (p<0.048) in tumor-bearing dogs (Table 2, Figure 7). These results show that a CD11b^low^/CADO48A^low^ myeloid precursor population was increased in tumor-bearing dogs. We next determined whether tumor-type influenced the levels of CD11b^low^/CADO48A^low^ cells. Table 3 demonstrates the percentage of CD11b^+^CADO48A^+^ cell populations when gated on all live cells (R1), all myeloid non-lymphocyte cells (P1) and CD11b/CADO48A subpopulations (P2-P4) across individual tumor types (i.e. sarcoma, carcinoma and melanoma). Table 3 shows an increased percentage of CD11b^+^CADO48A^+^ cells in both all live cells (i.e. R1 gate, p=0.027) and in the myeloid non-lymphocytes (i.e. P1 gate, p=0.036). When comparing across tumor types compared to controls with melanoma-bearing dogs demonstrating the highest levels of R1 and P1. However, an increase in P4 was not consistently evident when samples were grouped on individual tumor-types. Figure 6 demonstrates representative plots of CD11b/CADO48A expression in control dogs and dogs with differing tumor types. Taken together, these data demonstrate that CADO48A^low^/CD11b^low^ cells were increased in tumor-bearing dogs and that melanoma-bearing dogs demonstrate the highest levels of CD11b^+^CADO48A^+^ cells compared to either sarcomas or carcinomas.

**Table 1:** Description of patient characteristics for the control and experimental groups

***Control***		
Median Age, years		5
Sex		
	Male	19
	Female	21
Breed		
	Mixed	17
	Greyhound	13
	Labrador Retriever	3
	Boxer	2
	Collie	2
	Other	3
*Tumor*		
Median Age, years		9
Sex		
	Male	23
	Female	17
Breed		
	Lab Retriever	6
	Mixed	6
	Greyhound	4
	Golden Retriever	4
	Beagle	3
	Other	17
Tumor Type		
	Sarcoma	19
	Carcinoma	18
	Oral Melanoma	3

Patient characteristics at the time of study enrollment are described.

**Table 2:** Comparison of percent of cells positive for CD11b/CADO48A by gate of control and tumor-bearing dogs

***Control***	**Mean**	**Median**	**Min**	**Max**
Ungated	31.9±14.4	27.2	12.6	58.2
R1	28.4±10.3	24.4	10.9	50.6
P1	27.5±10.1	23.6	10.6	49.0
P2	24.2±09.2	20.8	8.6	45.3
P3	4.6±2.8	4.0	1.5	13.4
P4	3.6±1.7*	3.0	1.3	7.7
***Tumor***				
Ungated	33.7±15.0	29.3	11.4	72.3
R1	30.0±14.1	26.9	8.5	66.9
P1	29.1±14.0	26.0	7.2	65.2
P2	25.5±12.8	22.5	8.1	59.6
P3	4.5±3.6	3.5	1.2	20.3
P4	7.9±3.0*	3.7	1.1	12.7

The percentage of cells staining positive for CD11b and CADO48A are displayed for each gate analyzed for both the control and tumor-bearing populations. Cells from the P4 gate of the tumor bearing dogs were found to be elevated as compared to the healthy control dogs. No other statistical differences were seen in the other gates analyzed. An * denotes statistical significance (P<0.05). All values are expressed as a percentage of all cells analyzed by flow cytometry.

**Table 3:** Comparison of percent of cells positive for CD11b/CADO48A by gate amongst tumor types

***Sarcoma***	**Mean**	**Median**	**Min**	**Max**
Ungated	33.4±12.7	28.7	13.5	58.2
R1	29.9±12.5*	26.5	11.7	53.1
P1	29.0±12.6*	25.5	11.6	51.8
P2	25.1±12.0	22.4	8.2	50.6
P3	4.8±4.7	3.5	1.6	20.3
P4	4.7±2.9	3.8	1.1	12.6
***Carcinoma***				
Ungated	30.4±14.8	23.8	11.4	64.5
R1	26.4±12.8*	21.5	8.5	55.6
P1	25.3±12.5*	21.0	7.2	53.6
P2	22.7±11.4	18.2	8.1	47.9
P3	3.7±1.9	3.1	1.2	8.0
P4	5.1±3.5	3.5	2.1	12.7
***Melanoma***				
Ungated	56.0±15.2	53.5	42.3	72.3
R1	52.6±13.5*	51.0	40.0	66.9
P1	51.6±13.0*	50.2	39.3	65.2
P2	44.7±13.5	41.2	33.3	59.6
P3	7.8±2.2	7.7	5.7	10.1
P4	4.8±1.1	4.5	3.8	6.0

The percentage of cells staining positive for CD11b and CADO48A are displayed for each tumor subtype: sarcoma (n= 19), carcinoma (n=18) and melanoma (n=3). Patients were categorized into one of the three groups based on either cytology or histopathology. A statistical difference was seen in the R1 and P1 gates between the 3 tumor types on initial analysis. When the melanoma patients were excluded due to low patient numbers no statistical difference was seen in the percent of CD11b/CADO48A positive cells, in any gate, between the sarcoma or carcinoma patients. An * denoted statistical significance (p<0.05). All values are expressed as a percentage of all cells analyzed by flow cytometry.

### Suppressive abilities of CD11b^low^/CADO48A^low^ cells

We next wanted to determine whether the increase in CD11b^low^/CADO48A^low^ cells present in tumor-bearing dogs could impact immune function. To do this, we generated CD11b^low^/CADO48A^low^ cells and assessed their ability to suppress the proliferation of responder canine immune cells. To generate sufficient numbers of CD11b^low^/CADO48A^low^ cells for functional evaluation, we utilized an *in vitro* system to differentiate canine MDSCs and control myeloid precursor cells from bone marrow myeloid progenitors. Sorted CD11b^low^/CADO48A^low^ cells were able to suppress the proliferation of responder canine splenocytes following conA stimulation but not in LPS-stimulated cells (Figure 7). Cellular morphology was relatively similar between control and melanoma myeloid precursors although overall expression levels of both CD11b and CADO48A was slightly decreased in the myeloid cells exposed to melanoma tumor-conditioned media which is consistent with the relative arrest in differentiation described for MDSCs. Taken together, these results show that CD11b^low^/CADO48A^low^ cells were functionally immunosuppressive and likely represent a canine MDSC population.

## Discussion

This study investigated potential canine-specific markers for identifying MDSCs in the peripheral blood of dogs with cancer. We began our studies by first identifying potential phenotypic markers that could be used for identifying canine MDSCs. Prototypic MDSC phenotypic markers have been reported as CD11b^+^/CD33^+^/HLDR^low^ in humans and CD11b^+^/Gr-1^+^ in mice [1]. In humans, CD15 (granulocytic) and CD14 (monocytic) identify subpopulations of these cells while, in mice, CD11b^+^Ly6G^+^ and CD11b^+^Ly6C^+^ cells identify these cells as either granulocytic or monocytic, respectively. Unfortunately, the lack of readily available commercial canine antibodies has limited the identification of MDSCs and MDSC subsets in dogs.

CD11b, an integrin, is found on a variety of cells of myeloid origin and has been used as one phenotypic marker of MDSCs present in the mouse and man. In the dog, CD11b in known to be a marker of cells of myeloid origin, most specifically neutrophils [23]. There is a commercially available anti-canine CD11b available which has been validated. In the mouse, Gr-1 is a granulocytic marker whose co-expression with CD11b is used to identify the immature myeloid cell population that represents MDSCs. Although CD11b is useful to identify myeloid (e.g. macrophages/DCs), neutrophils and mast cells in the dog, no Gr-1 equivalent for the dog has been described. Additionally, murine Gr-1 has demonstrated a variable ability to stain canine cells, and its individual components, Ly6G and Ly6C, are not reported to cross-react (unpublished observations) [21,24,25].

In man, an equivalent antibody to Gr-1 does not exist. Rather, CD33, CD14 and HLA-DR (i.e. MHC class II) expression are used to specifically identify these cells. No canine specific, or cross-reacting, CD33 antibody is currently commercially available. Although human CD14 cross-reactive with canine cells, canine CD14 expression is variable on monocytes and likely may not truly mimic what is seen in humans [26]. Additionally, standardized protocols and antibody concentrations using CD14 in the canine have not been established [27]. However, a recent publication by Goulart et al. has demonstrated the use of negative labeling for CD14 expression to identify an MDSC population in the dog but no studies have investigated canine-specific granulocytic antibodies to identify an MDSCs in the dog [21]. Considering the lack of anti-canine CD33 and human MDSC subpopulations express markers which are not commercially available (e.g. canine-specific CD15 or CD14), we chose to evaluate the levels of canine-specific CD11b and a granulocytic marker which models the phenotypic description for murine MDSCs.

Only two commercially available markers are reported to label canine granulocytes (i.e. CADO48A and DH59A) and our results show that CADO48A is able to distinguish separate populations that are not evident in DH59A stained populations from canine peripheral blood. While our focus was on canine-specific antibodies, we did evaluate the cross-reactivity of murine Gr-1 with canine peripheral blood samples. Murine Gr-1 (clone RB6-8C5) failed to stain canine peripheral blood cells, results which differ from the ability of this clone to stain canine cells by Goulart et al. [21]. Our studies suggest that canine-specific CADO48A is an effective antibody to identify circulating myeloid cells in canine blood.

The ability of CADO48A to identify individual granulocytic populations of peripheral white blood cells (high versus low expression) as seen in Figure 1 suggests that this antibody may be useful for identifying granulocytic versus monocytic subpopulation but purification and functional evaluation of such subpopulations were beyond the scope of this study. Our results demonstrate that CD11b and CADO48 staining is useful for detecting subpopulations of myeloid cells in the peripheral blood of dogs. From our optimization studies, we found that clinical samples can be stored up to 24 hours in EDTA, 48 hours in media and that cells that have been fixed following staining with flow antibodies can be analyzed up to 72 hours later without dramatically impacting the expression of CD11b^+^CADO48^+^ levels. From a diagnostic standpoint, it is important to know the relative expression levels of these markers depending on sample treatment and handling.

Following optimization, we next went on to determine whether tumor-bearing dogs demonstrated altered levels of myeloid cell populations. Our results show that a specific population of CD11b^low^CADO48A^low^ cells was increased in tumor-bearing dogs. The finding of an upregulated population of cells in tumor-bearing dogs expressing CD11b, as well as, the surrogate marker CADO48A, is suggestive of an MDSC phenotype in the canine. Classically, MDSC expression of CD11b is considered to be high. In our canine samples, we found that the expression in the canine patient to be low. While this finding is discordant from the literature on MDSCs in the mouse and human, CD11b expression is known to be variable both in healthy people at different time points and in animals with varying severity of inflammation [28,29]. Additionally, it has been shown in human cell lines that during neutrophil maturation from a promyelocytic stage of development to a more terminally differentiated state CD11b expression starts low and eventually reaches high levels of expression once fully mature [30]. If the cells in our tumor-bearing dogs represent an immature myeloid population, low CD11b expression may be representative of MDSCs. Additionally, work done by Furuhashi et al. has shown that DCs with high CD11b expression are more capable of eliciting a T-cell response in the pulmonary parenchyma of mice, indicating that increased CD11b expression is a relative marker of increased maturation and/or activation of DCs [31]. Based on our results and previously published findings, low CD11b expression may in fact be indicative of an early myeloid derived cell population such as MDSC. Additionally, the cellular morphology of our purified CD11b^low^CADO48A^low^ cells is consistent with an immature and heterogeneous cellular morphology described for MDSC populations [1,18,19] in mice which contain cells demonstrating monocytic, granulocytic and ring-shaped nuclear forms (Figure 7B). Thus, we postulated that CD11b^low^CADO48A^low^ represent an MDSC population.

In order to determine whether, indeed, CD11b^low^CADO48A^low^ were immunosuppressive, we utilized our *in vitro* model to generate canine MDSCs under the influence of a tumor environment and purified the CD11b^low^CADO48A^low^ population. As Figure 7 demonstrates, CD11b^low^CADO48A^low^ were able to suppress the proliferation of responder canine lymphocytes. While cell sorting and functional evaluation of individual cancer patients were beyond the scope of this study, our data from purified CD11b^low^CADO48A^low^ (from control or tumor environment) verified that CD11b^low^CADO48A^low^ were immunosuppressive. Ongoing studies are evaluating the functional abilities and signaling pathways involved in the development of canine MDSCs using our *in vitro* MDSCs model.

While significant differences in R1 and P1 were found when tumor-bearing patients were grouped according to tumor type (Table 3), it was somewhat surprising that P4 differences were not significant. The most likely reason for this is that subcategorization decreased the number of patients in each tumor type category which then resulted in a lack of statitistical significance. An additional prospective study with recruitment of more patients according to individual tumor types would likely address this possibility. One interesting aspect of our study is the mixed population of tumors evaluated. In the human literature, the predominance of tumors shown to upregulate MDSCs are carcinomas, including those arising from the pancreas, colon and lung [32-34]. Unlike in humans, sarcomas are a relatively common tumor type seen in veterinary species and studies investigating MDSC populations in humans have found this cell type primarily in various carcinomas and melanoma [35]. The fact that MDSCs were upregulated in our tumor-bearing population, regardless of tumor type suggests that common mechanisms exist between sarcomas and carcinomas for the induction of MDSCs in the canine population. Interestingly, melanomas appear to upregulate MDSCs regardless of species (Table 3) [32-35]. Studies of MDSCs in dogs may be useful at dissecting the potential mechanisms by which carcinomas, sarcomas or melanomas differentially regulate MDSCs levels and why these may differ between species. Additionally, specific tumor types may uniquely upregulate specific MDSC subsets such as seen with the predominance of CD15^+^ MDSC or lineage negative MDSCs in patients with glioblastoma [36]. At present, a detailed evaluated of canine MDSC subpopulations is not feasible but our data suggest that CADO48A may be a useful antibody for such distinctions.

CD11b^+^CD14^-^MHCII^-^ cells have been identified as MDSCs in the peripheral blood of dogs (Goulart 2012) in both solitary and metastatic cancer and these cells were able to suppress T cell proliferation similar to what we found with CD11b^low^CADO48A^low^ cells [115]. Our studies demonstrate that CADO48A is a useful marker to identify MDSCs in dogs and it is not known whether CD11b^low^CADO48A^low^ and CD11b^+^CD14^-^MHCII^-^ represent two distinct MDSC populations (e.g. granulocytic and monocytic, respectively) and, if so, whether these two populations have distinct functional profiles. The identification of MDSC subpopulations is likely to be useful for diagnostic and prognostic purposes but the characterization of these populations in canines is only in its infancy. Additional markers, such as S100A9, have been described to identify monocytic MDSCs in humans with colon cancer and cross-reacting S100A9 proteins are available [33]. Ongoing studies evaluating various MDSC markers are needed to determine the phenotype and function of specific MDSC subpopulations (e.g. monocytic and granulocytic) in dogs. Utilization of both canine patients and an *in vitro* model system as we provide here are likely to provide important data on MDSC phenotype and function and facilitate translational applications between canine and human MDSC studies.

## Conclusion

This study identified canine specific markers that can be used to identify specific myeloid cell populations within clinical samples from dogs. Our data demonstrate that canine-specific antibodies can be used to identify a specific population of myeloid cells (CD11b^low^CADO48A^low^) which are increased in tumor-bearing canine patients and that purified CD11b^low^CADO48A^low^ cells suppressed the proliferation of canine lymphocytes. This work provides a foundation for future investigations of MDSC levels which may serve as a prognostic indicator in canine patients and help guide translational research approaches for cancer immunotherapy in human and veterinary cancer patients. The field of immunotherapy, both in man and in the veterinary field, is in a state of constant discovery. The ability to identify and monitor MDSC levels in the dog will be useful in the development and evaluation of new therapies in both man and his best friend.

## Competing interests

The authors declare that they have no competing interests that could inappropriately influence or bias the content of this paper.

## Authors’ contribution

MS assisted in the design of the study, conducted the experiments, interpreted the data and drafted the manuscript. WK participated in study design and helped to draft the manuscript. CL participated in study design and helped to draft the manuscript. SOM performed statistical analysis. TP conceived the study, participate in its design and helped to draft the manuscript. All authors read and approved the final manuscript.
